# 41-Azido-41-de­oxy­rapamycin

**DOI:** 10.1107/S1600536812012548

**Published:** 2012-03-28

**Authors:** Lijun Xie, Jie Huang, Jian Zuo, Hui Yu, Yuanrong Cheng

**Affiliations:** a Fujian Institute of Microbiology, Fuzhou, Fujian 350007, People’s Republic of China; b Key Laboratory of Marine Chemistry Theory and Technology, Ministry of Education, College of Chemistry and Chemical Engineering, Ocean University of China, Qingdao, Shandong 266100, People’s Republic of China

## Abstract

The title compound, C_51_H_78_N_4_O_12_, is a derivative of rapamycin, a triene macrolide anti­biotic mol­ecule isolated from *Streptomyces hygroscopicus*. The macrocyclic ring structure has 15 chiral centres, with one of the substituent hy­droxy groups giving an intra­molecular hydrogen bond to a ketone O-atom acceptor. The mol­ecules also form inter­molecular hy­droxy–ketone O—H⋯O hydrogen-bonding associations, giving one-dimensional chains extending along (010). The crystal has 108 Å^3^ solvent-accessible voids.

## Related literature

For general background on rapamycin, as an immunosuppressant drug for rejection prevention in organ transplantation, see: Calne *et al.* (1989 ▶). For the anti­cancer properties of rapamycin derivatives, see: Chan (2004 ▶); Sun *et al.* (2005 ▶); Ayral-Kaloustian *et al.* (2010 ▶). For the structure of rapamycin, see: White & Swindells (1981 ▶); Findlay & Radics (1980 ▶). For related literature, see: Flack (1983 ▶).
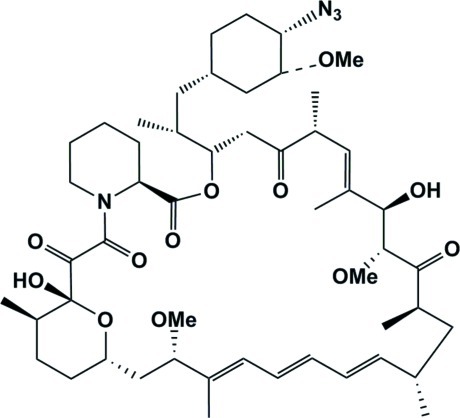


## Experimental

### 

#### Crystal data

C_51_H_78_N_4_O_12_
*M* *_r_* = 939.17
Orthorhombic, 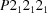

*a* = 12.7461 (7) Å
*b* = 12.9824 (7) Å
*c* = 34.4022 (12) Å
*V* = 5692.7 (5) Å^3^
*Z* = 4
Cu *K*α radiation
μ = 0.63 mm^−1^
*T* = 293 K
0.42 × 0.30 × 0.25 mm

#### Data collection

Bruker SMART CCD area-detector diffractometer
Absorption correction: multi-scan (*SADABS*; Sheldrick, 1996 ▶) *T*_min_ = 0.778, *T*_max_ = 0.858
29197 measured reflections
8388 independent reflections
4624 reflections with *I* > 2σ(*I*)
*R*_int_ = 0.049
θ_max_ = 60.0°

#### Refinement

*R*[*F* ^2^ > 2σ(*F* ^2^)] = 0.093
*wR*(*F* ^2^) = 0.331
*S* = 1.09
8388 reflections
615 parameters
H-atom parameters constrained
Δρ_max_ = 0.33 e Å^−3^
Δρ_min_ = −0.30 e Å^−3^

### 

Data collection: *SMART* (Bruker, 1996 ▶); cell refinement: *SMART*; data reduction: *SAINT* (Bruker, 1996 ▶); program(s) used to solve structure: *SHELXS97* (Sheldrick, 2008 ▶); program(s) used to refine structure: *SHELXL97* (Sheldrick, 2008 ▶); molecular graphics: *SHELXTL* (Sheldrick, 2008 ▶); software used to prepare material for publication: *SHELXTL*.

## Supplementary Material

Crystal structure: contains datablock(s) I, global. DOI: 10.1107/S1600536812012548/zs2189sup1.cif

Structure factors: contains datablock(s) I. DOI: 10.1107/S1600536812012548/zs2189Isup2.hkl

Additional supplementary materials:  crystallographic information; 3D view; checkCIF report

## Figures and Tables

**Table 1:** Hydrogen-bond geometry (Å, °)

*D*—H⋯*A*	*D*—H	H⋯*A*	*D*⋯*A*	*D*—H⋯*A*
O2—H2⋯O6^i^	0.82	2.08	2.891 (9)	172
O8—H8⋯O10	0.82	2.40	3.060 (13)	138

Symmetry code: (i) 
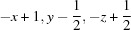
.

**Fig. 1.**
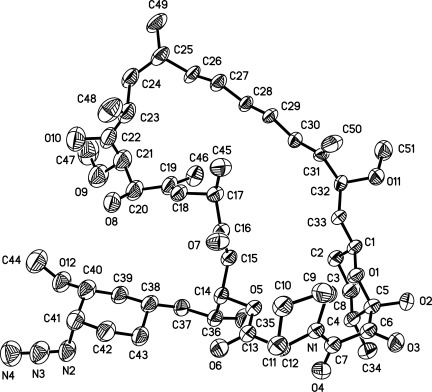
The structure of the title compound, showing 30% probability displacement ellipsoids and the atom-numbering scheme. Hydrogen atoms are omitted.

## supplementary crystallographic information

### Comment

Rapamycin is a triene macrolide antibiotic isolated from *Streptomyces hygroscopicus*, with anti-fungal, anti-proliferative, immunosuppressive and anti-tumor activities. Currently, rapamycin is used as immunosuppressant in organ transplantation and as drug-eluting stents in chronic heart disease (Calne *et al.*, 1989). In recent years, interest has focused on its potential as an anti-tumor drug. Rapamycin analogs Everolimus (RAD-001) and Temsirolimus (CCI-779) have been used in clinical trials. In addition, Deferolimus (AP-23537), has been in clinical trials as a potent anticancer agent (Chan, 2004; Sun *et al.*, 2005; Ayral-Kaloustian *et al.*, 2010). Herein, we present the synthesis and structure of a rapamycin derivative, the title compound (41-azido-41-deoxy)rapamycin, C_51_H_78_N_4_O_12_. The structure of rapamycin has previously been reported (White & Swindells, 1981; Findlay & Radics, 1980).

The structure of the title compound, shown in Fig. 1 has a 31 atom macrocyclic ring structure having 15 chiral centres and comprises an oxygen bridge between C1 and C5, an amide C7—N1 bond, a lactone C13—O5, and an additional bond between N1 and C12 which forms a piperidine unit. One of the substituent hydroxy groups gives an intramolecular hydrogen bond to a ketone O-acceptor while in the crystal structure there are 108 Å^3^ solvent accessible voids with adjacent molecules forming intermolecular hydroxy O—H···O_ketone_hydrogen-bonding interactions (Table 1), giving one-dimensional chains extending along (010). The absolute configuration for the rapamycin molecular framework was not determined.

### Experimental

Trifluoromethanesulfonic anhydride (10.7 mmol, 1.8 ml) was added gradually to a solution of rapamycin (5.5 mmol, 5 g) and 2,6-lutidine (32.7 mmol, 3.8 ml) in dry dichloromethane (30 ml) and the reaction mixture was stirred at 273 K for 1 h. The mixture was quenched with a saturated NaHCO_3_ solution (100 ml) and diluted with dichloromethane. The two phases were separated and the organic phase was washed with brine, and was then collected and dried over anhydrous Na_2_SO_4_ overnight. After filtering, the solvent was evaporated under reduced pressure to obtain the product which was dissolved in a mixture of acetone and distilled water (100/1, *v*/*v*). Sodium azide (22 mmol, 1.4 g) was added and the mixture was stirred at room temperature for 6 h, then concentrated under reduced pressure. The crude mixture was purified by silica gel column chromatography (1:5 EtOAc/hexanes) to obtain the title compound as a white powder. Crystals suitable for X-ray analysis were grown by slow room temperature evaporation of a solution in ether.

### Refinement

All H-atoms were positioned geometrically and refined using a riding model, with O—H = 0.82 Å, C—H = 0.96 Å (CH_3_), 0.97 Å (CH_2_) and 0.98 Å (CH), with *U*_iso_(H) = 1.2*U*_eq_(C, O). In the absence of a suitable heavy atom in the molecule, the absolute configuration for the rapamycin molecular framework, which has 15 chiral centres was not determined [absolute structure factor (Flack, 1983): -0.03 (5) for 3646 Friedel pairs].

### Crystal data

**Table:**

C_51_H_78_N_4_O_12_	*F*(000) = 2032
*M**_r_* = 939.17	*D*_x_ = 1.096 Mg m^−^^3^
Orthorhombic, *P*2_1_2_1_2_1_	Cu *K*α radiation, λ = 1.54184 Å
Hall symbol: P 2ac 2ab	Cell parameters from 5478 reflections
*a* = 12.7461 (7) Å	θ = 2.6–71.8°
*b* = 12.9824 (7) Å	µ = 0.63 mm^−^^1^
*c* = 34.4022 (12) Å	*T* = 293 K
*V* = 5692.7 (5) Å^3^	Block, colorless
*Z* = 4	0.42 × 0.30 × 0.25 mm

### Data collection

**Table:**

Bruker SMART CCD area-detector diffractometer	8388 independent reflections
Radiation source: fine-focus sealed tube	4624 reflections with *I* > 2σ(*I*)
Graphite monochromator	*R*_int_ = 0.049
φ and ω scans	θ_max_ = 60.0°, θ_min_ = 2.6°
Absorption correction: multi-scan (*SADABS*; Sheldrick, 1996)	*h* = −13→14
*T*_min_ = 0.778, *T*_max_ = 0.858	*k* = −14→14
29197 measured reflections	*l* = −26→38

### Refinement

**Table:**

Refinement on *F*^2^	Secondary atom site location: difference Fourier map
Least-squares matrix: full	Hydrogen site location: inferred from neighbouring sites
*R*[*F*^2^ > 2σ(*F*^2^)] = 0.093	H-atom parameters constrained
*wR*(*F*^2^) = 0.331	*w* = 1/[σ^2^(*F*_o_^2^) + (0.1701*P*)^2^ + 1.7435*P*] where *P* = (*F*_o_^2^ + 2*F*_c_^2^)/3
*S* = 1.09	(Δ/σ)_max_ = 0.001
8388 reflections	Δρ_max_ = 0.33 e Å^−^^3^
615 parameters	Δρ_min_ = −0.30 e Å^−^^3^
0 restraints	Extinction correction: *SHELXL97* (Sheldrick, 2008), Fc^*^=kFc[1+0.001xFc^2^λ^3^/sin(2θ)]^-1/4^
Primary atom site location: structure-invariant direct methods	Extinction coefficient: 0.00017 (17)

### Special details

**Table:**

Geometry. All e.s.d.'s (except the e.s.d. in the dihedral angle between two l.s. planes) are estimated using the full covariance matrix. The cell e.s.d.'s are taken into account individually in the estimation of e.s.d.'s in distances, angles and torsion angles; correlations between e.s.d.'s in cell parameters are only used when they are defined by crystal symmetry. An approximate (isotropic) treatment of cell e.s.d.'s is used for estimating e.s.d.'s involving l.s. planes.
Refinement. Refinement of *F*^2^ against ALL reflections. The weighted *R*-factor *wR* and goodness of fit *S* are based on *F*^2^, conventional *R*-factors *R* are based on *F*, with *F* set to zero for negative *F*^2^. The threshold expression of *F*^2^ > σ(*F*^2^) is used only for calculating *R*-factors(gt) *etc*. and is not relevant to the choice of reflections for refinement. *R*-factors based on *F*^2^ are statistically about twice as large as those based on *F*, and *R*- factors based on ALL data will be even larger.

### Fractional atomic coordinates and isotropic or equivalent isotropic displacement parameters (Å^2^)

**Table:**

	*x*	*y*	*z*	*U*_iso_*/*U*_eq_	
N1	0.4521 (6)	0.3588 (5)	0.25753 (16)	0.1026 (18)	
N2	0.3802 (9)	0.8666 (8)	0.4697 (3)	0.150 (3)	
N3	0.3528 (10)	0.9168 (10)	0.4926 (4)	0.172 (4)	
N4	0.3321 (13)	0.9709 (11)	0.5184 (4)	0.217 (5)	
O1	0.6698 (4)	0.2159 (4)	0.26859 (12)	0.0994 (14)	
O2	0.7631 (5)	0.2089 (4)	0.21010 (14)	0.1135 (16)	
H2	0.7235	0.1651	0.2012	0.136*	
O3	0.5771 (6)	0.2783 (5)	0.18713 (16)	0.145 (2)	
O4	0.5614 (6)	0.4776 (5)	0.23361 (18)	0.1327 (19)	
O5	0.4895 (4)	0.4333 (4)	0.32932 (12)	0.1033 (14)	
O6	0.3584 (5)	0.5464 (5)	0.32564 (16)	0.1284 (19)	
O7	0.3845 (6)	0.3104 (5)	0.39436 (19)	0.136 (2)	
O8	0.4701 (7)	0.4427 (7)	0.53139 (19)	0.167 (3)	
H8	0.4453	0.4351	0.5532	0.201*	
O9	0.6484 (9)	0.4613 (10)	0.5890 (3)	0.208 (4)	
O10	0.4991 (9)	0.3651 (9)	0.6147 (3)	0.211 (4)	
O11	0.7906 (5)	−0.0375 (5)	0.32119 (14)	0.1200 (18)	
O12	0.4698 (7)	0.7458 (6)	0.5318 (2)	0.165 (3)	
C1	0.7506 (6)	0.1683 (6)	0.29287 (18)	0.096 (2)	
H1	0.7903	0.1190	0.2771	0.115*	
C2	0.8252 (7)	0.2503 (7)	0.3079 (2)	0.108 (2)	
H2A	0.8824	0.2179	0.3219	0.130*	
H2B	0.7883	0.2956	0.3257	0.130*	
C3	0.8688 (8)	0.3127 (8)	0.2741 (2)	0.123 (3)	
H3A	0.9118	0.2685	0.2579	0.147*	
H3B	0.9131	0.3673	0.2841	0.147*	
C4	0.7811 (7)	0.3601 (7)	0.2492 (2)	0.114 (2)	
H4	0.7401	0.4065	0.2657	0.137*	
C5	0.7082 (7)	0.2711 (6)	0.2358 (2)	0.107 (2)	
C6	0.6055 (7)	0.3084 (7)	0.2192 (2)	0.109 (2)	
C7	0.5380 (8)	0.3880 (7)	0.2384 (2)	0.111 (2)	
C8	0.4334 (8)	0.2488 (7)	0.2691 (3)	0.121 (3)	
H8A	0.4584	0.2379	0.2954	0.145*	
H8B	0.4726	0.2036	0.2519	0.145*	
C9	0.3176 (9)	0.2224 (8)	0.2668 (3)	0.137 (3)	
H9A	0.2964	0.2194	0.2398	0.164*	
H9B	0.3063	0.1548	0.2781	0.164*	
C10	0.2502 (8)	0.3001 (8)	0.2878 (3)	0.128 (3)	
H10A	0.1767	0.2815	0.2854	0.154*	
H10B	0.2680	0.3014	0.3152	0.154*	
C11	0.2686 (8)	0.4013 (8)	0.2705 (3)	0.127 (3)	
H11A	0.2243	0.4512	0.2836	0.152*	
H11B	0.2472	0.3990	0.2435	0.152*	
C12	0.3817 (7)	0.4388 (6)	0.2724 (2)	0.106 (2)	
H12	0.3872	0.4977	0.2547	0.127*	
C13	0.4102 (8)	0.4779 (7)	0.3130 (2)	0.107 (2)	
C14	0.5112 (7)	0.4722 (6)	0.36905 (18)	0.104 (2)	
H14	0.4451	0.4926	0.3814	0.125*	
C15	0.5559 (7)	0.3802 (6)	0.3899 (2)	0.103 (2)	
H15A	0.5996	0.4044	0.4111	0.124*	
H15B	0.6006	0.3427	0.3720	0.124*	
C16	0.4750 (8)	0.3069 (7)	0.4061 (2)	0.111 (2)	
C17	0.5093 (7)	0.2379 (7)	0.4392 (2)	0.105 (2)	
H17	0.5812	0.2143	0.4343	0.126*	
C18	0.5081 (9)	0.3024 (7)	0.4751 (2)	0.123 (3)	
H18	0.4436	0.3318	0.4812	0.147*	
C19	0.5808 (9)	0.3229 (9)	0.4984 (2)	0.132 (3)	
C20	0.5758 (11)	0.4023 (11)	0.5306 (3)	0.156 (4)	
H20	0.6226	0.4590	0.5233	0.187*	
C21	0.6134 (13)	0.3609 (13)	0.5684 (4)	0.174 (5)	
H21	0.6741	0.3159	0.5642	0.209*	
C22	0.5386 (12)	0.3100 (15)	0.5872 (3)	0.160 (5)	
C23	0.5082 (11)	0.2036 (13)	0.5795 (3)	0.154 (4)	
H23	0.5294	0.1876	0.5528	0.185*	
C24	0.5708 (8)	0.1253 (11)	0.6081 (2)	0.146 (4)	
H24A	0.6394	0.1545	0.6134	0.176*	
H24B	0.5333	0.1216	0.6325	0.176*	
C25	0.5857 (8)	0.0181 (10)	0.5931 (2)	0.135 (3)	
H25	0.5172	−0.0104	0.5859	0.161*	
C26	0.6556 (7)	0.0189 (8)	0.55779 (17)	0.119 (3)	
H26	0.7228	0.0451	0.5615	0.143*	
C27	0.6341 (7)	−0.0126 (8)	0.52235 (18)	0.114 (3)	
H27	0.5712	−0.0480	0.5192	0.137*	
C28	0.6956 (6)	0.0011 (6)	0.48806 (16)	0.0928 (18)	
H28	0.7634	0.0263	0.4913	0.111*	
C29	0.6651 (6)	−0.0188 (6)	0.45253 (16)	0.0949 (19)	
H29	0.6012	−0.0526	0.4499	0.114*	
C30	0.7184 (6)	0.0054 (6)	0.41760 (17)	0.0950 (19)	
H30	0.7827	0.0380	0.4212	0.114*	
C31	0.6929 (6)	−0.0101 (5)	0.38036 (16)	0.0889 (18)	
C32	0.7555 (6)	0.0366 (6)	0.34803 (16)	0.094 (2)	
H32	0.8161	0.0728	0.3590	0.113*	
C33	0.6918 (6)	0.1111 (6)	0.32327 (17)	0.095 (2)	
H33A	0.6361	0.0727	0.3107	0.114*	
H33B	0.6590	0.1609	0.3404	0.114*	
C34	0.8238 (9)	0.4220 (8)	0.2155 (3)	0.144 (3)	
H34A	0.7669	0.4546	0.2020	0.216*	
H34B	0.8606	0.3771	0.1980	0.216*	
H34C	0.8711	0.4737	0.2251	0.216*	
C35	0.6878 (8)	0.5458 (8)	0.3477 (3)	0.135 (3)	
H35A	0.6766	0.5217	0.3217	0.203*	
H35B	0.7258	0.4947	0.3621	0.203*	
H35C	0.7275	0.6086	0.3471	0.203*	
C36	0.5840 (7)	0.5650 (7)	0.3669 (2)	0.110 (2)	
H36	0.5479	0.6171	0.3512	0.132*	
C37	0.6006 (8)	0.6127 (7)	0.4078 (2)	0.115 (3)	
H37A	0.6467	0.5676	0.4224	0.138*	
H37B	0.6366	0.6779	0.4047	0.138*	
C38	0.5016 (9)	0.6314 (7)	0.4321 (2)	0.121 (3)	
H38	0.4696	0.5642	0.4374	0.145*	
C39	0.5282 (9)	0.6808 (7)	0.4709 (2)	0.129 (3)	
H39A	0.5776	0.6372	0.4846	0.155*	
H39B	0.5620	0.7466	0.4662	0.155*	
C40	0.4340 (11)	0.6975 (9)	0.4960 (3)	0.142 (4)	
H40	0.4055	0.6297	0.5027	0.170*	
C41	0.3477 (11)	0.7584 (9)	0.4768 (3)	0.142 (3)	
H41	0.2846	0.7570	0.4930	0.170*	
C42	0.3241 (10)	0.7134 (9)	0.4374 (3)	0.147 (4)	
H42A	0.2756	0.7587	0.4240	0.176*	
H42B	0.2893	0.6476	0.4409	0.176*	
C43	0.4199 (8)	0.6975 (8)	0.4120 (3)	0.129 (3)	
H43A	0.3989	0.6646	0.3879	0.155*	
H43B	0.4503	0.7638	0.4056	0.155*	
C44	0.4192 (14)	0.7247 (13)	0.5642 (4)	0.213 (7)	
H44A	0.4238	0.6523	0.5695	0.320*	
H44B	0.3469	0.7441	0.5615	0.320*	
H44C	0.4501	0.7625	0.5853	0.320*	
C45	0.4390 (10)	0.1460 (9)	0.4428 (3)	0.153 (4)	
H45A	0.3671	0.1680	0.4425	0.230*	
H45B	0.4534	0.1111	0.4669	0.230*	
H45C	0.4514	0.1000	0.4215	0.230*	
C46	0.6913 (8)	0.2799 (10)	0.4921 (3)	0.144 (4)	
H46A	0.6957	0.2491	0.4668	0.217*	
H46B	0.7060	0.2288	0.5116	0.217*	
H46C	0.7415	0.3348	0.4941	0.217*	
C47	0.7008 (16)	0.4346 (18)	0.6248 (5)	0.253 (9)	
H47A	0.7625	0.3950	0.6191	0.380*	
H47B	0.6544	0.3947	0.6408	0.380*	
H47C	0.7204	0.4964	0.6383	0.380*	
C48	0.3956 (11)	0.1988 (15)	0.5810 (4)	0.201 (7)	
H48A	0.3671	0.2665	0.5773	0.302*	
H48B	0.3740	0.1726	0.6058	0.302*	
H48C	0.3705	0.1540	0.5608	0.302*	
C49	0.6345 (11)	−0.0495 (11)	0.6259 (2)	0.182 (5)	
H49A	0.6960	−0.0162	0.6360	0.273*	
H49B	0.6533	−0.1156	0.6155	0.273*	
H49C	0.5842	−0.0584	0.6464	0.273*	
C50	0.5983 (9)	−0.0713 (8)	0.3692 (3)	0.141 (3)	
H50A	0.5447	−0.0257	0.3598	0.212*	
H50B	0.5727	−0.1081	0.3914	0.212*	
H50C	0.6167	−0.1194	0.3491	0.212*	
C51	0.8704 (10)	−0.1028 (10)	0.3372 (3)	0.156 (4)	
H51A	0.8383	−0.1566	0.3522	0.234*	
H51B	0.9158	−0.0629	0.3536	0.234*	
H51C	0.9106	−0.1327	0.3165	0.234*	

### Atomic displacement parameters (Å^2^)

**Table:**

	*U*^11^	*U*^22^	*U*^33^	*U*^12^	*U*^13^	*U*^23^
N1	0.139 (5)	0.095 (4)	0.074 (3)	0.007 (4)	−0.010 (4)	0.005 (3)
N2	0.201 (9)	0.128 (7)	0.122 (6)	−0.035 (6)	0.035 (6)	−0.033 (5)
N3	0.210 (10)	0.159 (10)	0.147 (8)	−0.041 (8)	0.026 (8)	−0.026 (7)
N4	0.259 (14)	0.186 (12)	0.207 (12)	−0.014 (11)	0.038 (11)	−0.038 (10)
O1	0.121 (3)	0.106 (3)	0.071 (2)	−0.003 (3)	−0.005 (2)	0.015 (2)
O2	0.144 (4)	0.114 (4)	0.083 (3)	0.011 (3)	0.019 (3)	−0.010 (3)
O3	0.198 (6)	0.152 (5)	0.085 (3)	0.055 (5)	−0.035 (4)	−0.020 (3)
O4	0.169 (5)	0.101 (4)	0.127 (4)	−0.002 (4)	0.020 (4)	0.015 (3)
O5	0.134 (4)	0.105 (3)	0.070 (2)	0.011 (3)	−0.005 (3)	0.003 (2)
O6	0.153 (5)	0.132 (5)	0.101 (3)	0.030 (4)	−0.012 (3)	−0.002 (3)
O7	0.142 (5)	0.137 (5)	0.129 (4)	−0.003 (4)	−0.034 (4)	0.032 (4)
O8	0.206 (7)	0.186 (7)	0.110 (4)	0.065 (6)	0.014 (4)	0.000 (4)
O9	0.243 (9)	0.250 (11)	0.133 (6)	0.021 (9)	−0.008 (6)	−0.015 (7)
O10	0.242 (9)	0.236 (10)	0.154 (7)	0.044 (9)	0.024 (7)	−0.024 (7)
O11	0.158 (5)	0.117 (4)	0.085 (3)	0.026 (4)	−0.017 (3)	−0.004 (3)
O12	0.235 (8)	0.137 (5)	0.124 (5)	−0.043 (6)	0.032 (5)	−0.022 (4)
C1	0.129 (5)	0.096 (5)	0.062 (3)	−0.004 (4)	−0.001 (4)	0.008 (3)
C2	0.133 (6)	0.115 (5)	0.077 (4)	0.000 (5)	−0.011 (4)	0.001 (4)
C3	0.148 (7)	0.122 (6)	0.098 (5)	−0.023 (6)	−0.008 (5)	0.005 (5)
C4	0.151 (7)	0.113 (6)	0.077 (4)	−0.010 (5)	0.009 (4)	0.013 (4)
C5	0.136 (6)	0.107 (5)	0.076 (4)	0.006 (5)	−0.006 (4)	0.012 (4)
C6	0.142 (7)	0.109 (5)	0.076 (4)	0.002 (5)	−0.010 (4)	0.010 (4)
C7	0.146 (7)	0.104 (6)	0.084 (4)	0.002 (6)	−0.006 (5)	0.003 (4)
C8	0.147 (8)	0.111 (6)	0.105 (5)	0.004 (6)	−0.015 (5)	0.005 (5)
C9	0.160 (9)	0.118 (7)	0.132 (7)	0.006 (7)	−0.012 (6)	0.007 (6)
C10	0.144 (7)	0.130 (7)	0.110 (5)	−0.002 (6)	−0.001 (5)	0.001 (6)
C11	0.155 (8)	0.129 (7)	0.097 (5)	0.007 (6)	−0.017 (5)	0.001 (5)
C12	0.138 (7)	0.098 (5)	0.082 (4)	0.012 (5)	−0.005 (4)	0.011 (4)
C13	0.136 (7)	0.106 (6)	0.079 (4)	0.007 (5)	0.000 (5)	0.001 (4)
C14	0.139 (6)	0.106 (5)	0.068 (3)	−0.001 (5)	0.002 (4)	−0.010 (3)
C15	0.130 (6)	0.109 (5)	0.071 (4)	−0.004 (5)	−0.002 (4)	0.002 (4)
C16	0.133 (7)	0.125 (6)	0.075 (4)	0.015 (6)	−0.013 (4)	0.001 (4)
C17	0.118 (5)	0.111 (5)	0.085 (4)	0.009 (5)	0.001 (4)	0.013 (4)
C18	0.147 (7)	0.141 (7)	0.079 (4)	0.034 (6)	0.010 (5)	0.019 (5)
C19	0.156 (8)	0.167 (9)	0.074 (4)	0.028 (7)	0.006 (5)	0.002 (5)
C20	0.185 (10)	0.195 (11)	0.088 (5)	0.028 (9)	−0.001 (6)	−0.003 (6)
C21	0.201 (13)	0.205 (14)	0.116 (8)	0.032 (11)	0.001 (9)	−0.005 (9)
C22	0.170 (11)	0.216 (14)	0.094 (6)	0.044 (11)	0.026 (7)	0.004 (8)
C23	0.166 (11)	0.211 (13)	0.085 (5)	0.037 (10)	0.012 (6)	0.011 (7)
C24	0.153 (8)	0.210 (12)	0.077 (4)	0.048 (8)	0.013 (5)	0.023 (6)
C25	0.138 (7)	0.200 (11)	0.065 (4)	0.020 (7)	−0.002 (4)	0.028 (5)
C26	0.123 (5)	0.178 (8)	0.057 (3)	0.028 (6)	0.014 (3)	0.027 (4)
C27	0.125 (5)	0.152 (7)	0.066 (4)	0.028 (6)	−0.001 (4)	0.017 (4)
C28	0.105 (4)	0.117 (5)	0.056 (3)	0.015 (4)	0.000 (3)	0.004 (3)
C29	0.116 (5)	0.110 (5)	0.059 (3)	0.008 (4)	0.004 (3)	0.016 (3)
C30	0.117 (5)	0.101 (5)	0.067 (3)	0.010 (4)	−0.004 (3)	−0.003 (3)
C31	0.122 (5)	0.084 (4)	0.061 (3)	−0.009 (4)	−0.015 (3)	0.012 (3)
C32	0.125 (5)	0.100 (5)	0.056 (3)	0.002 (4)	−0.012 (3)	−0.013 (3)
C33	0.126 (5)	0.100 (5)	0.060 (3)	0.004 (4)	0.001 (3)	0.009 (3)
C34	0.182 (9)	0.135 (7)	0.114 (6)	−0.010 (7)	0.021 (6)	0.027 (6)
C35	0.165 (8)	0.130 (7)	0.110 (6)	−0.030 (7)	0.037 (6)	−0.016 (5)
C36	0.145 (7)	0.107 (5)	0.078 (4)	−0.006 (5)	−0.002 (4)	−0.008 (4)
C37	0.157 (7)	0.099 (5)	0.089 (4)	−0.014 (5)	−0.007 (5)	−0.012 (4)
C38	0.174 (8)	0.106 (5)	0.083 (4)	−0.027 (6)	0.004 (5)	−0.004 (4)
C39	0.183 (9)	0.111 (6)	0.093 (5)	−0.021 (6)	0.009 (6)	−0.009 (5)
C40	0.197 (10)	0.122 (7)	0.106 (6)	−0.039 (8)	0.009 (7)	−0.022 (6)
C41	0.186 (10)	0.121 (7)	0.118 (7)	−0.035 (8)	0.032 (7)	−0.014 (6)
C42	0.184 (10)	0.135 (8)	0.122 (7)	−0.028 (7)	0.023 (7)	−0.008 (6)
C43	0.170 (8)	0.117 (6)	0.099 (5)	−0.021 (6)	0.017 (6)	−0.014 (5)
C44	0.293 (19)	0.190 (13)	0.157 (11)	−0.049 (14)	0.051 (12)	−0.019 (10)
C45	0.176 (9)	0.142 (8)	0.142 (8)	0.019 (8)	0.011 (7)	0.032 (7)
C46	0.135 (7)	0.182 (11)	0.116 (6)	0.003 (7)	0.000 (6)	−0.014 (7)
C47	0.283 (19)	0.31 (2)	0.168 (12)	0.026 (19)	−0.023 (14)	−0.055 (15)
C48	0.162 (11)	0.30 (2)	0.138 (9)	0.016 (12)	−0.015 (8)	0.038 (11)
C49	0.236 (12)	0.235 (14)	0.075 (4)	0.027 (11)	0.015 (6)	0.051 (7)
C50	0.182 (9)	0.141 (8)	0.101 (5)	−0.051 (7)	−0.017 (6)	0.025 (5)
C51	0.184 (10)	0.162 (9)	0.121 (7)	0.037 (9)	−0.013 (7)	−0.010 (7)

### Geometric parameters (Å, º)

**Table:**

N1—C7	1.332 (11)	C23—H23	0.9800
N1—C12	1.465 (10)	C24—C25	1.496 (15)
N1—C8	1.502 (11)	C24—H24A	0.9700
N2—N3	1.081 (13)	C24—H24B	0.9700
N2—C41	1.485 (14)	C25—C26	1.507 (11)
N3—N4	1.162 (15)	C25—C49	1.559 (13)
O1—C5	1.424 (9)	C25—H25	0.9800
O1—C1	1.463 (9)	C26—C27	1.315 (10)
O2—C5	1.387 (9)	C26—H26	0.9300
O2—H2	0.8200	C27—C28	1.428 (10)
O3—C6	1.226 (9)	C27—H27	0.9300
O4—C7	1.213 (10)	C28—C29	1.308 (9)
O5—C13	1.293 (10)	C28—H28	0.9300
O5—C14	1.484 (8)	C29—C30	1.416 (9)
O6—C13	1.189 (9)	C29—H29	0.9300
O7—C16	1.224 (10)	C30—C31	1.337 (8)
O8—C20	1.447 (14)	C30—H30	0.9300
O8—H8	0.8200	C31—C50	1.495 (11)
O9—C47	1.442 (19)	C31—C32	1.497 (9)
O9—C21	1.550 (18)	C32—C33	1.523 (10)
O10—C22	1.287 (15)	C32—H32	0.9800
O11—C32	1.406 (8)	C33—H33A	0.9700
O11—C51	1.435 (12)	C33—H33B	0.9700
O12—C44	1.317 (14)	C34—H34A	0.9600
O12—C40	1.456 (11)	C34—H34B	0.9600
C1—C33	1.485 (9)	C34—H34C	0.9600
C1—C2	1.517 (11)	C35—C36	1.499 (12)
C1—H1	0.9800	C35—H35A	0.9600
C2—C3	1.523 (11)	C35—H35B	0.9600
C2—H2A	0.9700	C35—H35C	0.9600
C2—H2B	0.9700	C36—C37	1.553 (10)
C3—C4	1.536 (12)	C36—H36	0.9800
C3—H3A	0.9700	C37—C38	1.532 (13)
C3—H3B	0.9700	C37—H37A	0.9700
C4—C34	1.512 (11)	C37—H37B	0.9700
C4—C5	1.552 (12)	C38—C43	1.517 (13)
C4—H4	0.9800	C38—C39	1.520 (11)
C5—C6	1.508 (12)	C38—H38	0.9800
C6—C7	1.498 (12)	C39—C40	1.494 (15)
C8—C9	1.517 (14)	C39—H39A	0.9700
C8—H8A	0.9700	C39—H39B	0.9700
C8—H8B	0.9700	C40—C41	1.507 (16)
C9—C10	1.509 (14)	C40—H40	0.9800
C9—H9A	0.9700	C41—C42	1.507 (13)
C9—H9B	0.9700	C41—H41	0.9800
C10—C11	1.463 (14)	C42—C43	1.517 (14)
C10—H10A	0.9700	C42—H42A	0.9700
C10—H10B	0.9700	C42—H42B	0.9700
C11—C12	1.523 (13)	C43—H43A	0.9700
C11—H11A	0.9700	C43—H43B	0.9700
C11—H11B	0.9700	C44—H44A	0.9600
C12—C13	1.530 (10)	C44—H44B	0.9600
C12—H12	0.9800	C44—H44C	0.9600
C14—C15	1.505 (11)	C45—H45A	0.9600
C14—C36	1.523 (11)	C45—H45B	0.9600
C14—H14	0.9800	C45—H45C	0.9600
C15—C16	1.511 (12)	C46—H46A	0.9600
C15—H15A	0.9700	C46—H46B	0.9600
C15—H15B	0.9700	C46—H46C	0.9600
C16—C17	1.513 (11)	C47—H47A	0.9600
C17—C18	1.493 (12)	C47—H47B	0.9600
C17—C45	1.497 (14)	C47—H47C	0.9600
C17—H17	0.9800	C48—H48A	0.9600
C18—C19	1.252 (13)	C48—H48B	0.9600
C18—H18	0.9300	C48—H48C	0.9600
C19—C20	1.515 (15)	C49—H49A	0.9600
C19—C46	1.530 (14)	C49—H49B	0.9600
C20—C21	1.486 (16)	C49—H49C	0.9600
C20—H20	0.9800	C50—H50A	0.9600
C21—C22	1.330 (19)	C50—H50B	0.9600
C21—H21	0.9800	C50—H50C	0.9600
C22—C23	1.459 (19)	C51—H51A	0.9600
C23—C48	1.437 (17)	C51—H51B	0.9600
C23—C24	1.623 (15)	C51—H51C	0.9600
			
C7—N1—C12	118.4 (7)	C49—C25—H25	109.0
C7—N1—C8	122.1 (7)	C27—C26—C25	128.5 (9)
C12—N1—C8	119.0 (7)	C27—C26—H26	115.7
N3—N2—C41	111.1 (10)	C25—C26—H26	115.7
N2—N3—N4	174.1 (18)	C26—C27—C28	127.8 (9)
C5—O1—C1	115.0 (6)	C26—C27—H27	116.1
C5—O2—H2	109.5	C28—C27—H27	116.1
C13—O5—C14	113.1 (6)	C29—C28—C27	125.7 (7)
C20—O8—H8	109.5	C29—C28—H28	117.1
C47—O9—C21	108.8 (13)	C27—C28—H28	117.1
C32—O11—C51	112.2 (6)	C28—C29—C30	127.3 (7)
C44—O12—C40	118.3 (10)	C28—C29—H29	116.3
O1—C1—C33	105.0 (6)	C30—C29—H29	116.3
O1—C1—C2	109.9 (6)	C31—C30—C29	131.6 (7)
C33—C1—C2	115.3 (6)	C31—C30—H30	114.2
O1—C1—H1	108.9	C29—C30—H30	114.2
C33—C1—H1	108.9	C30—C31—C50	121.5 (7)
C2—C1—H1	108.9	C30—C31—C32	121.4 (7)
C1—C2—C3	110.0 (6)	C50—C31—C32	117.0 (6)
C1—C2—H2A	109.7	O11—C32—C31	112.3 (6)
C3—C2—H2A	109.7	O11—C32—C33	103.7 (5)
C1—C2—H2B	109.7	C31—C32—C33	112.9 (6)
C3—C2—H2B	109.7	O11—C32—H32	109.2
H2A—C2—H2B	108.2	C31—C32—H32	109.2
C2—C3—C4	111.9 (7)	C33—C32—H32	109.2
C2—C3—H3A	109.2	C1—C33—C32	116.3 (6)
C4—C3—H3A	109.2	C1—C33—H33A	108.2
C2—C3—H3B	109.2	C32—C33—H33A	108.2
C4—C3—H3B	109.2	C1—C33—H33B	108.2
H3A—C3—H3B	107.9	C32—C33—H33B	108.2
C34—C4—C3	112.2 (8)	H33A—C33—H33B	107.4
C34—C4—C5	112.6 (7)	C4—C34—H34A	109.5
C3—C4—C5	107.6 (7)	C4—C34—H34B	109.5
C34—C4—H4	108.1	H34A—C34—H34B	109.5
C3—C4—H4	108.1	C4—C34—H34C	109.5
C5—C4—H4	108.1	H34A—C34—H34C	109.5
O2—C5—O1	112.6 (6)	H34B—C34—H34C	109.5
O2—C5—C6	112.5 (6)	C36—C35—H35A	109.5
O1—C5—C6	99.3 (6)	C36—C35—H35B	109.5
O2—C5—C4	108.7 (7)	H35A—C35—H35B	109.5
O1—C5—C4	110.2 (6)	C36—C35—H35C	109.5
C6—C5—C4	113.2 (7)	H35A—C35—H35C	109.5
O3—C6—C7	116.6 (8)	H35B—C35—H35C	109.5
O3—C6—C5	119.6 (8)	C35—C36—C14	115.3 (7)
C7—C6—C5	123.6 (7)	C35—C36—C37	110.2 (7)
O4—C7—N1	122.8 (9)	C14—C36—C37	110.7 (6)
O4—C7—C6	117.4 (9)	C35—C36—H36	106.7
N1—C7—C6	119.6 (8)	C14—C36—H36	106.7
N1—C8—C9	110.9 (8)	C37—C36—H36	106.7
N1—C8—H8A	109.5	C38—C37—C36	116.4 (7)
C9—C8—H8A	109.5	C38—C37—H37A	108.2
N1—C8—H8B	109.5	C36—C37—H37A	108.2
C9—C8—H8B	109.5	C38—C37—H37B	108.2
H8A—C8—H8B	108.1	C36—C37—H37B	108.2
C10—C9—C8	112.3 (9)	H37A—C37—H37B	107.3
C10—C9—H9A	109.1	C43—C38—C39	108.4 (8)
C8—C9—H9A	109.1	C43—C38—C37	114.0 (7)
C10—C9—H9B	109.1	C39—C38—C37	111.2 (8)
C8—C9—H9B	109.1	C43—C38—H38	107.7
H9A—C9—H9B	107.9	C39—C38—H38	107.7
C11—C10—C9	108.3 (8)	C37—C38—H38	107.7
C11—C10—H10A	110.0	C40—C39—C38	112.9 (9)
C9—C10—H10A	110.0	C40—C39—H39A	109.0
C11—C10—H10B	110.0	C38—C39—H39A	109.0
C9—C10—H10B	110.0	C40—C39—H39B	109.0
H10A—C10—H10B	108.4	C38—C39—H39B	109.0
C10—C11—C12	115.0 (8)	H39A—C39—H39B	107.8
C10—C11—H11A	108.5	O12—C40—C39	107.4 (9)
C12—C11—H11A	108.5	O12—C40—C41	111.9 (10)
C10—C11—H11B	108.5	C39—C40—C41	114.2 (9)
C12—C11—H11B	108.5	O12—C40—H40	107.7
H11A—C11—H11B	107.5	C39—C40—H40	107.7
N1—C12—C11	109.8 (7)	C41—C40—H40	107.7
N1—C12—C13	114.1 (7)	N2—C41—C42	106.0 (9)
C11—C12—C13	111.7 (7)	N2—C41—C40	111.4 (10)
N1—C12—H12	106.9	C42—C41—C40	109.6 (10)
C11—C12—H12	106.9	N2—C41—H41	109.9
C13—C12—H12	106.9	C42—C41—H41	109.9
O6—C13—O5	127.6 (7)	C40—C41—H41	109.9
O6—C13—C12	116.7 (8)	C41—C42—C43	114.3 (10)
O5—C13—C12	115.7 (8)	C41—C42—H42A	108.7
O5—C14—C15	103.9 (6)	C43—C42—H42A	108.7
O5—C14—C36	109.8 (6)	C41—C42—H42B	108.7
C15—C14—C36	114.8 (7)	C43—C42—H42B	108.7
O5—C14—H14	109.4	H42A—C42—H42B	107.6
C15—C14—H14	109.4	C38—C43—C42	111.5 (8)
C36—C14—H14	109.4	C38—C43—H43A	109.3
C14—C15—C16	114.7 (7)	C42—C43—H43A	109.3
C14—C15—H15A	108.6	C38—C43—H43B	109.3
C16—C15—H15A	108.6	C42—C43—H43B	109.3
C14—C15—H15B	108.6	H43A—C43—H43B	108.0
C16—C15—H15B	108.6	O12—C44—H44A	109.5
H15A—C15—H15B	107.6	O12—C44—H44B	109.5
O7—C16—C15	119.9 (8)	H44A—C44—H44B	109.5
O7—C16—C17	123.0 (9)	O12—C44—H44C	109.5
C15—C16—C17	117.0 (8)	H44A—C44—H44C	109.5
C18—C17—C45	111.8 (7)	H44B—C44—H44C	109.5
C18—C17—C16	106.7 (7)	C17—C45—H45A	109.5
C45—C17—C16	111.2 (8)	C17—C45—H45B	109.5
C18—C17—H17	109.0	H45A—C45—H45B	109.5
C45—C17—H17	109.0	C17—C45—H45C	109.5
C16—C17—H17	109.0	H45A—C45—H45C	109.5
C19—C18—C17	129.8 (9)	H45B—C45—H45C	109.5
C19—C18—H18	115.1	C19—C46—H46A	109.5
C17—C18—H18	115.1	C19—C46—H46B	109.5
C18—C19—C20	125.5 (10)	H46A—C46—H46B	109.5
C18—C19—C46	120.9 (9)	C19—C46—H46C	109.5
C20—C19—C46	112.9 (10)	H46A—C46—H46C	109.5
O8—C20—C21	114.5 (10)	H46B—C46—H46C	109.5
O8—C20—C19	107.4 (9)	O9—C47—H47A	109.5
C21—C20—C19	112.3 (11)	O9—C47—H47B	109.5
O8—C20—H20	107.4	H47A—C47—H47B	109.5
C21—C20—H20	107.4	O9—C47—H47C	109.5
C19—C20—H20	107.4	H47A—C47—H47C	109.5
C22—C21—C20	112.1 (13)	H47B—C47—H47C	109.5
C22—C21—O9	113.6 (13)	C23—C48—H48A	109.5
C20—C21—O9	101.0 (12)	C23—C48—H48B	109.5
C22—C21—H21	110.0	H48A—C48—H48B	109.5
C20—C21—H21	110.0	C23—C48—H48C	109.5
O9—C21—H21	110.0	H48A—C48—H48C	109.5
O10—C22—C21	111.2 (17)	H48B—C48—H48C	109.5
O10—C22—C23	123.8 (14)	C25—C49—H49A	109.5
C21—C22—C23	124.9 (13)	C25—C49—H49B	109.5
C48—C23—C22	107.4 (14)	H49A—C49—H49B	109.5
C48—C23—C24	116.3 (12)	C25—C49—H49C	109.5
C22—C23—C24	110.6 (11)	H49A—C49—H49C	109.5
C48—C23—H23	107.4	H49B—C49—H49C	109.5
C22—C23—H23	107.4	C31—C50—H50A	109.5
C24—C23—H23	107.4	C31—C50—H50B	109.5
C25—C24—C23	115.9 (9)	H50A—C50—H50B	109.5
C25—C24—H24A	108.3	C31—C50—H50C	109.5
C23—C24—H24A	108.3	H50A—C50—H50C	109.5
C25—C24—H24B	108.3	H50B—C50—H50C	109.5
C23—C24—H24B	108.3	O11—C51—H51A	109.5
H24A—C24—H24B	107.4	O11—C51—H51B	109.5
C24—C25—C26	110.2 (9)	H51A—C51—H51B	109.5
C24—C25—C49	109.0 (8)	O11—C51—H51C	109.5
C26—C25—C49	110.6 (9)	H51A—C51—H51C	109.5
C24—C25—H25	109.0	H51B—C51—H51C	109.5
C26—C25—H25	109.0		
			
C5—O1—C1—C33	−176.8 (6)	C18—C19—C20—C21	131.6 (13)
C5—O1—C1—C2	58.7 (7)	C46—C19—C20—C21	−57.8 (15)
O1—C1—C2—C3	−54.0 (8)	O8—C20—C21—C22	39.2 (19)
C33—C1—C2—C3	−172.3 (7)	C19—C20—C21—C22	−83.7 (16)
C1—C2—C3—C4	55.6 (10)	O8—C20—C21—O9	−82.0 (14)
C2—C3—C4—C34	−179.9 (8)	C19—C20—C21—O9	155.1 (11)
C2—C3—C4—C5	−55.5 (9)	C47—O9—C21—C22	67.4 (16)
C1—O1—C5—O2	61.5 (8)	C47—O9—C21—C20	−172.4 (12)
C1—O1—C5—C6	−179.2 (6)	C20—C21—C22—O10	−102.0 (14)
C1—O1—C5—C4	−60.1 (8)	O9—C21—C22—O10	11.7 (16)
C34—C4—C5—O2	56.4 (9)	C20—C21—C22—C23	81.1 (17)
C3—C4—C5—O2	−67.8 (7)	O9—C21—C22—C23	−165.2 (11)
C34—C4—C5—O1	−179.6 (8)	O10—C22—C23—C48	45.2 (16)
C3—C4—C5—O1	56.2 (9)	C21—C22—C23—C48	−138.3 (13)
C34—C4—C5—C6	−69.4 (10)	O10—C22—C23—C24	−82.6 (15)
C3—C4—C5—C6	166.4 (6)	C21—C22—C23—C24	93.9 (14)
O2—C5—C6—O3	3.6 (11)	C48—C23—C24—C25	83.2 (15)
O1—C5—C6—O3	−115.7 (8)	C22—C23—C24—C25	−153.9 (10)
C4—C5—C6—O3	127.4 (8)	C23—C24—C25—C26	65.3 (12)
O2—C5—C6—C7	−171.2 (8)	C23—C24—C25—C49	−173.1 (9)
O1—C5—C6—C7	69.4 (9)	C24—C25—C26—C27	−119.9 (12)
C4—C5—C6—C7	−47.4 (10)	C49—C25—C26—C27	119.5 (11)
C12—N1—C7—O4	1.6 (11)	C25—C26—C27—C28	170.8 (9)
C8—N1—C7—O4	−169.5 (8)	C26—C27—C28—C29	−170.0 (9)
C12—N1—C7—C6	−173.5 (6)	C27—C28—C29—C30	171.5 (8)
C8—N1—C7—C6	15.3 (10)	C28—C29—C30—C31	−178.8 (8)
O3—C6—C7—O4	−93.5 (11)	C29—C30—C31—C50	−6.7 (13)
C5—C6—C7—O4	81.5 (10)	C29—C30—C31—C32	170.8 (8)
O3—C6—C7—N1	82.0 (10)	C51—O11—C32—C31	−70.2 (9)
C5—C6—C7—N1	−103.1 (9)	C51—O11—C32—C33	167.6 (7)
C7—N1—C8—C9	−145.2 (8)	C30—C31—C32—O11	125.4 (7)
C12—N1—C8—C9	43.7 (10)	C50—C31—C32—O11	−57.1 (9)
N1—C8—C9—C10	−50.2 (10)	C30—C31—C32—C33	−117.8 (7)
C8—C9—C10—C11	58.6 (11)	C50—C31—C32—C33	59.8 (9)
C9—C10—C11—C12	−59.4 (10)	O1—C1—C33—C32	168.3 (6)
C7—N1—C12—C11	146.1 (7)	C2—C1—C33—C32	−70.7 (9)
C8—N1—C12—C11	−42.5 (8)	O11—C32—C33—C1	−63.7 (8)
C7—N1—C12—C13	−87.6 (8)	C31—C32—C33—C1	174.4 (6)
C8—N1—C12—C13	83.8 (9)	O5—C14—C36—C35	−58.7 (9)
C10—C11—C12—N1	50.8 (10)	C15—C14—C36—C35	57.9 (9)
C10—C11—C12—C13	−76.8 (10)	O5—C14—C36—C37	175.3 (7)
C14—O5—C13—O6	3.1 (12)	C15—C14—C36—C37	−68.1 (9)
C14—O5—C13—C12	−178.2 (7)	C35—C36—C37—C38	−176.8 (8)
N1—C12—C13—O6	176.5 (7)	C14—C36—C37—C38	−47.9 (10)
C11—C12—C13—O6	−58.3 (10)	C36—C37—C38—C43	−55.5 (11)
N1—C12—C13—O5	−2.4 (10)	C36—C37—C38—C39	−178.4 (7)
C11—C12—C13—O5	122.9 (8)	C43—C38—C39—C40	55.7 (11)
C13—O5—C14—C15	148.9 (7)	C37—C38—C39—C40	−178.3 (9)
C13—O5—C14—C36	−87.9 (8)	C44—O12—C40—C39	−147.8 (12)
O5—C14—C15—C16	−83.4 (8)	C44—O12—C40—C41	86.2 (14)
C36—C14—C15—C16	156.8 (6)	C38—C39—C40—O12	−179.5 (8)
C14—C15—C16—O7	17.2 (11)	C38—C39—C40—C41	−54.8 (12)
C14—C15—C16—C17	−157.6 (7)	N3—N2—C41—C42	144.1 (13)
O7—C16—C17—C18	−96.2 (10)	N3—N2—C41—C40	−96.7 (13)
C15—C16—C17—C18	78.4 (9)	O12—C40—C41—N2	55.3 (11)
O7—C16—C17—C45	26.0 (12)	C39—C40—C41—N2	−67.0 (11)
C15—C16—C17—C45	−159.4 (8)	O12—C40—C41—C42	172.2 (8)
C45—C17—C18—C19	116.7 (12)	C39—C40—C41—C42	50.0 (12)
C16—C17—C18—C19	−121.5 (12)	N2—C41—C42—C43	69.7 (13)
C17—C18—C19—C20	170.0 (9)	C40—C41—C42—C43	−50.6 (12)
C17—C18—C19—C46	0.1 (18)	C39—C38—C43—C42	−55.5 (10)
C18—C19—C20—O8	4.8 (16)	C37—C38—C43—C42	−179.9 (7)
C46—C19—C20—O8	175.4 (9)	C41—C42—C43—C38	55.6 (12)

### Hydrogen-bond geometry (Å, º)

**Table:**

*D*—H···*A*	*D*—H	H···*A*	*D*···*A*	*D*—H···*A*
O2—H2···O6^i^	0.82	2.08	2.891 (9)	172
O8—H8···O10	0.82	2.40	3.060 (13)	138

Symmetry code: (i) −*x*+1, *y*−1/2, −*z*+1/2.

## References

[bb1] Ayral-Kaloustian, S., Gu, J., Lucas, J., Cinque, M., Gaydos, C., Zask, A., Chaudhary, I., Wang, J., Di, L., Young, M., Ruppen, M., Mansour, T. S., Gibbons, J. J. & Yu, K. (2010). *J. Med. Chem.* **53**, 452–459. 10.1021/jm901427g 19928864

[bb2] Bruker (1996). *SMART* and *SAINT* Bruker AXS Inc., Madison, Wisconsin, USA.

[bb4] Calne, R. Y., Collier, D. S., Lim, S., Pollard, S. G., Samaan, A., White, D. J. & Thiru, S. (1989). *Lancet*, **120**, 443–444. 10.1016/s0140-6736(89)90417-0 2568561

[bb5] Chan, S. (2004). *Br. J. Cancer*, **91**, 1420–1424. 10.1038/sj.bjc.6602162 PMC2409926 15365568

[bb6] Findlay, J. A. & Radics, L. (1980). *Can. J. Chem.* **58**, 579–590.

[bb7] Flack, H. D. (1983). *Acta Cryst.* A**39**, 876–881.

[bb8] Sheldrick, G. M. (1996). *SADABS* University of Göttingen, Germany.

[bb9] Sheldrick, G. M. (2008). *Acta Cryst.* A**64**, 112–122. 10.1107/S0108767307043930 18156677

[bb10] Sun, S. Y., Rosenberg, L. M., Wang, X., Zhou, Z. M., Yue, P., Fu, H. & Khuri, F. R. (2005). *Cancer Res.* **65**, 7052–7058. 10.1158/0008-5472.CAN-05-0917 16103051

[bb11] White, P. S. & Swindells, D. C. N. (1981). *Acta Cryst.* A**37**, C75–C76.

